# Opening the Strands of Replication Origins—Still an Open Question

**DOI:** 10.3389/fmolb.2016.00062

**Published:** 2016-09-30

**Authors:** Jyoti K. Jha, Revathy Ramachandran, Dhruba K. Chattoraj

**Affiliations:** Laboratory of Biochemistry and Molecular Biology, Center for Cancer Research, National Cancer Institute, National Institutes of HealthBethesda, MD, USA

**Keywords:** replication origins, DNA melting, bacterial origins, lambda origin, plasmid origins

## Abstract

The local separation of duplex DNA strands (strand opening) is necessary for initiating basic transactions on DNA such as transcription, replication, and homologous recombination. Strand opening is commonly a stage at which these processes are regulated. Many different mechanisms are used to open the DNA duplex, the details of which are of great current interest. In this review, we focus on a few well-studied cases of DNA replication origin opening in bacteria. In particular, we discuss the opening of origins that support the theta (θ) mode of replication, which is used by all chromosomal origins and many extra-chromosomal elements such as plasmids and phages. Although the details of opening can vary among different origins, a common theme is binding of the initiator to multiple sites at the origin, causing stress that opens an adjacent and intrinsically unstable A+T rich region. The initiator stabilizes the opening by capturing one of the open strands. How the initiator binding energy is harnessed for strand opening remains to be understood.

## Introduction

A remarkable feature of double stranded DNA (dsDNA) is its ability to undergo denaturation, whereby its strands can be completely separated into single strands, and renaturation, whereby the two complementary strands can be annealed back to form dsDNA. *In vitro*, DNA can undergo denaturation or renaturation simply in response to a change in salt concentration, temperature, pH or the presence of mild reagents such as formamide (Inman, 1966; Westmoreland et al., 1969). The reversibility of strand separation is the basis of hybridization techniques such as Southern blotting and PCR.

Strand opening usually refers to situations where the stability of duplex DNA is altered locally and for a limited period by DNA binding proteins. Complementary strands of DNA are most stable in the double helical B-form as modeled by Watson and Crick. Opening of the strands is thus energetically unfavorable. Active processes are involved in making the opening site-specific, and of significant length and duration so that the downstream events become feasible. In the case of replication initiation, the immediate downstream event is the loading of the replicative helicase. The helicase enlarges the opening and mediates loading of the primase and the replisome machinery that are required for duplicating the DNA (Bell and Kaguni, 2013).

**Figure 1:**
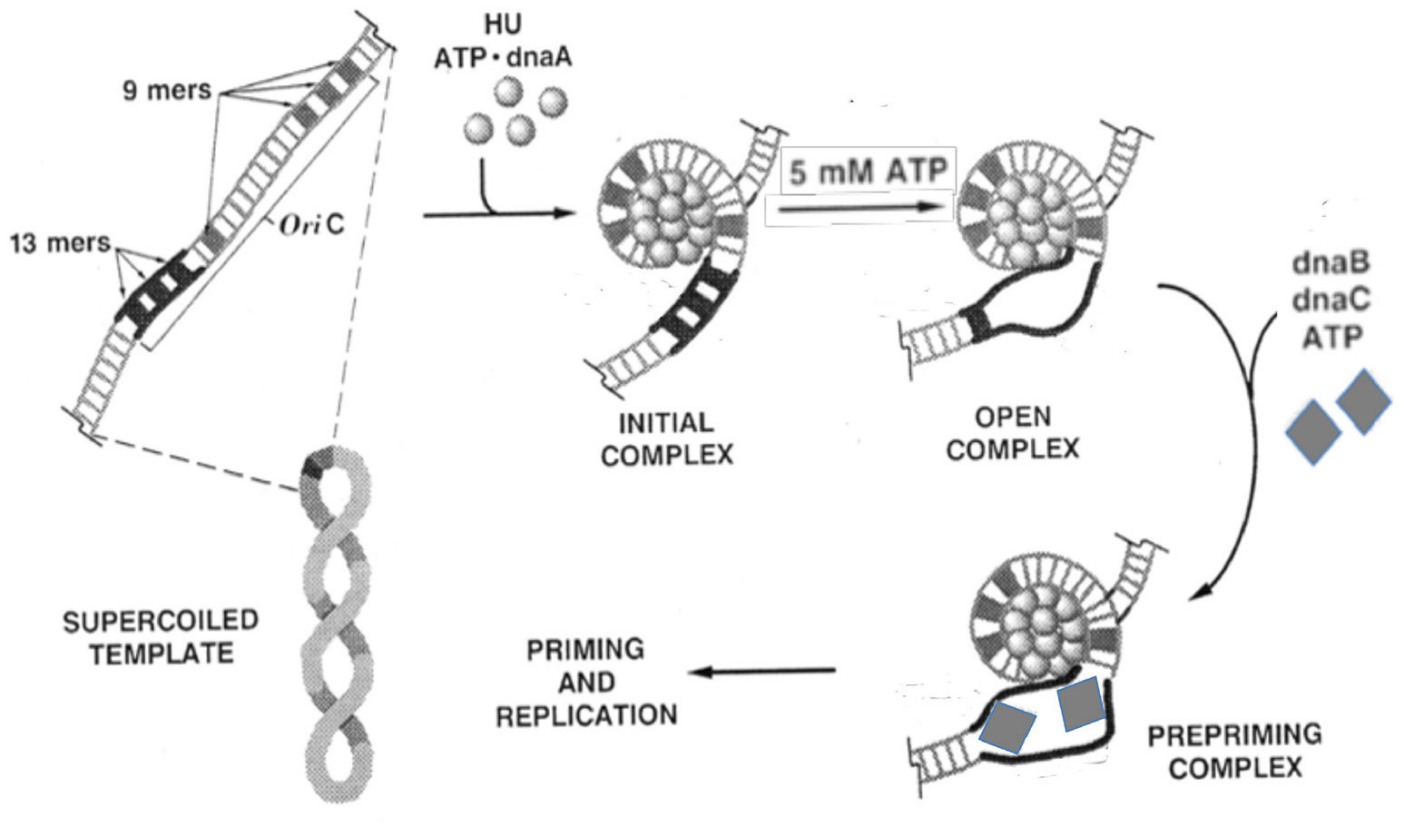
**Biochemical steps leading to initiation of replication from the *E. coli* origin, *oriC***. The figure is adapted from (Bramhill and Kornberg, 1988a) with permission from the publisher. The initiator DnaA initially binds to the 9-mers (R sites), and then saturates the origin in the presence of ATP and HU. Under favorable reaction conditions (high ATP, negative supercoiling, 38°C, appropriate concentration of HU), the region with three 13-mers (DUE) opens. The opening allows loading of the DnaB-DnaC complex (the helicase and the helicase loader), and subsequently the rest of the replisome to initiate and complete duplication of the minichromosome. Note that (i) the entire process can be achieved with purified components (Kaguni and Kornberg, 1984; Bramhill and Kornberg, 1988a); (ii) the top strand of the open region contacts the DnaA-ATP bundle, which stabilizes the open complex; (iii) DnaA-ATP binding to *oriC* can be achieved at < 1 μM concentrations of ATP, whereas the opening requires mM concentrations of ATP, most likely for reasons other than DNA binding (Saxena et al., 2015); (iv) The DNA spiral (“writhing”) shown surrounding the DnaA-ATP bundle is left-handed. Current evidence suggests it to be right-handed, which is more logical in terms of opening (Erzberger et al., 2006); and (v) HU stimulates opening, and helps to localize it to 13-mers, but is not essential (Bramhill and Kornberg, 1988a).

Among different origins, the structure and the process of strand opening vary significantly, but there are several commonalities (Bramhill and Kornberg, 1988b; Figure 1). Common elements include: (1) The presence of multiple initiator protein binding sites (9-mers) within the origin. The binding of the initiator allows site-specific opening, which enables helicase loading. (2) The presence of A+T-rich DNA sequences (13-mers) within the origin where the opening initiates. A stretch of ~20 bp A+T-rich region (called a DNA unwinding element, or DUE) is common within replication origins, most likely due to the fact that A+T-rich regions are easier to melt than G+C rich sequences (Inman, 1966; Kowalski and Eddy, 1989). (3) Remodeling (bending/folding/stretching) of origin DNA upon initiator binding, which is often facilitated by additional binding of nucleoid associated proteins (NAPs, e.g., HU; Stenzel et al., 1987; Hwang and Kornberg, 1992a; Dorman, 2009). (4) A requirement for the DNA to be negatively supercoiled, which is an under-wound and unstable state, that can make the DNA prone to opening but not open enough for helicase loading (Bramhill and Kornberg, 1988a). (5) Opening at DUE resulting from its intrinsic instability, and stress from DNA remodeling and negative supercoiling (Bowater et al., 1991). (6) Stabilization of the open state by the single stranded DNA (ssDNA) binding activity of the initiator, which captures one specific single strand of the open DNA so that the other is available for helicase loading. It is worth emphasizing that *in vivo* the aggregate of the A+T rich DUE, NAPs and negative supercoiling are not enough, and that the initiator binding to the origin provides an essential contribution to the energetics of opening. Additional regulatory factors are usually involved to modulate the frequency and timing of opening. Below we elaborate on the core features of opening for a few specific origins.

## Opening of an AAA+ protein controlled origin, *oriC*, of *Escherichia coli*

The opening of the *E. coli* origin, *oriC*, has been studied in the most depth. The opening was demonstrated *in vitro* at a time when DNA replication could be separated into discrete stages, with each step dependent on the previous one: initiator binding to the origin, strand opening at DUE, loading of the helicase, and finally, loading of the primase and the rest of the replisome (Figure 1; Bramhill and Kornberg, 1988b; O'Donnell, 2006). The ability to delineate the replication initiation process into discrete stages revealed that origin opening is not only a critical first step, but also a key replicon-specific event, as the players in subsequent steps seem common to all replicons.

**Figure 2:**
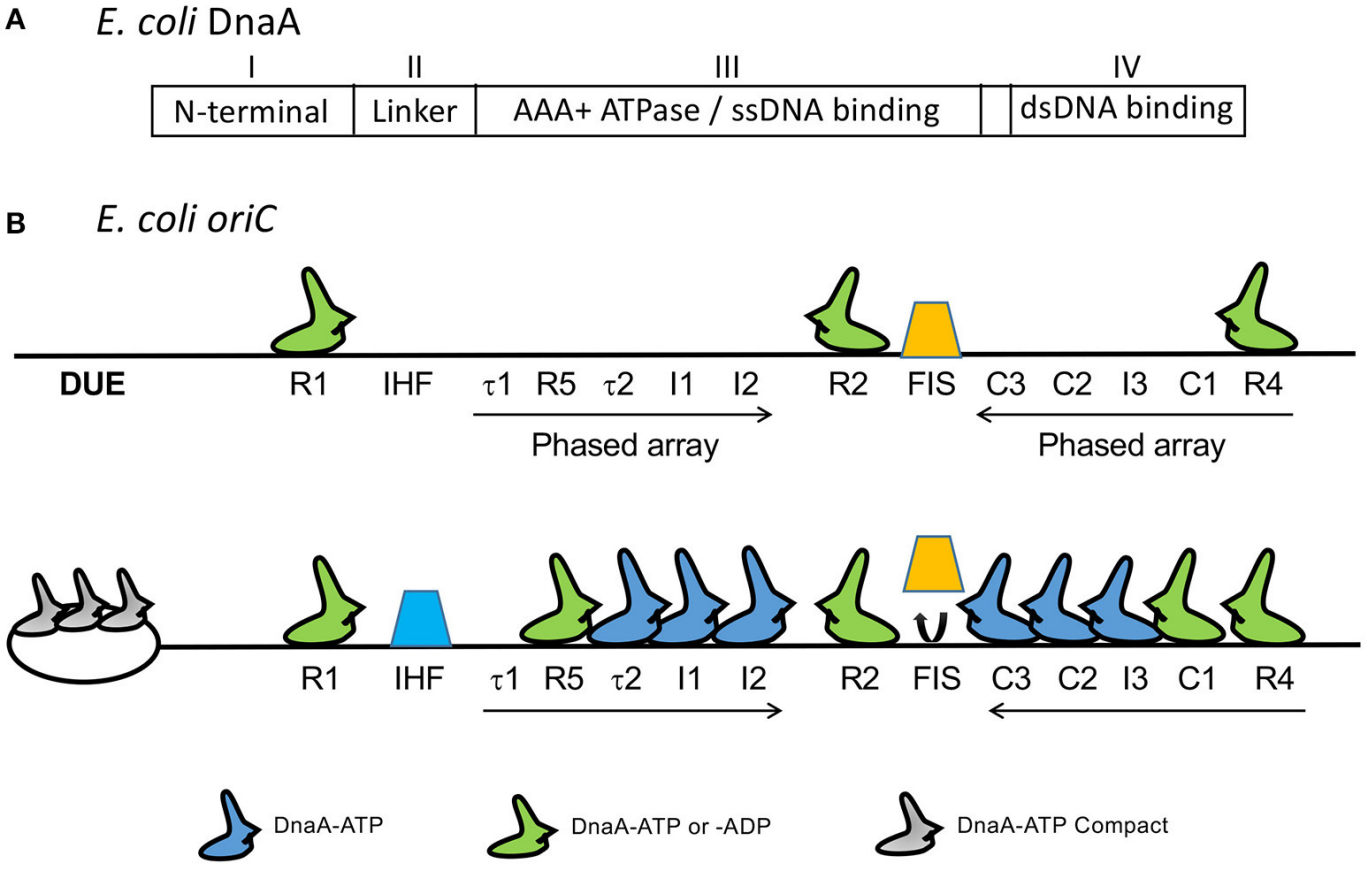
**(A)** The four domains of DnaA. **(B)** Map of *oriC* showing several DnaA binding sites that were identified subsequent to the 9-mers shown in Figure 1. The figure is adapted from (Rozgaja et al., 2011) with permission from the publisher. Three of the 9-mers are high-affinity R sites (R1, R2, and R4) that bind DnaA-ADP and DnaA-ATP with nearly equal affinity and they remain bound throughout the cell cycle. The remaining sites R5, τ1, τ2, I1-I3, and C1-C3 are present in two phased arrays and preferentially or exclusively bind to DnaA-ATP, except for R5 which binds both DnaA-ADP and DnaA-ATP, as other R sites do (McGarry et al., 2004; Kawakami et al., 2005; Rozgaja et al., 2011). [Note that DnaA binding site R3 is not shown. The identification of R3 could have been a misinterpretation of binding to C2 and C3, which overlap with R3 (Rozgaja et al., 2011)]. Upon accumulation of DnaA-ATP, the R1 and R4 sites nucleate sequential binding of DnaA to the two arrays. The DnaA oligomer extension could displace Fis (orange trapezoid), possibly removing some steric hindrance to IHF binding (blue trapezoid; Kaur et al., 2014).

Decades of genetic, biochemical and structural studies have generated a wealth of information on the structure-function relationship of the *E. coli* initiator, DnaA. DnaA is a highly conserved initiator protein in bacteria with structural similarity to initiators in the other domains of life (Giraldo, 2003). DnaA belongs to the AAA+ superfamily of ATPases (Neuwald et al., 1999) and has four domains (Ozaki and Katayama, 2009; Figure 2A): An N-terminal domain for homo-oligomerization and interactions with other replication related proteins, a non-conserved linker domain between the N-terminal domain and the large AAA+ domain for binding and hydrolyzing ATP, and a C-terminal DnaA binding domain (DBD) containing a helix-turn-helix (HTH) motif and a proximal basic loop for specific binding to dsDNA (Erzberger et al., 2002). The AAA+ domain mediates ATP dependent DnaA oligomerization that is independent of the N-terminal domain, which allows the AAA+ domain to bind to ssDNA (Duderstadt et al., 2011). DnaA thus uses two different domains to bind to ds- and ss-DNA. DnaA has several binding sites in *oriC*. The organization of the sites and their interactions with DnaA are complex (Leonard and Grimwade, 2010). Models have been proposed to explain how these interactions may give rise to strand opening, as we discuss below.

### Formation of nucleoprotein complexes at *oriC* of *E. coli*

DnaA binds through its C-terminal HTH motif to ds*oriC* at eleven 9-mer sites (Figure 2B). The three high affinity sites (R1, R2, and R4, Kd < 20 nM) remain bound throughout the cell cycle, and have equal affinity for DnaA-ATP and DnaA-ADP (Nievera et al., 2006). Binding to the remaining sites requires cooperative interactions with DnaA bound to the high affinity sites, with most requiring higher concentrations of DnaA-ATP. Binding to these weaker sites is cell-cycle specific and peaks immediately before the time of initiation, when the DnaA-ATP concentration reaches a maximum (Kurokawa et al., 1999; Nievera et al., 2006).

Two NAPs, Fis and IHF, regulate the timing of DNA-ATP binding. When bound to *oriC*, Fis inhibits saturation of DnaA-ATP binding to the weaker sites. Upon release of Fis, IHF binding facilitates saturation of binding (Ryan et al., 2004). These studies indicate that saturation of binding is a highly regulated process in the cell cycle, and is achieved by controlling the DnaA-ATP concentration. The increase in the DnaA-ATP concentration promotes oligomerization of DnaA-ATP from R4 to C3, which is believed to cause dissociation of Fis from its site that overlaps C3 (Rozgaja et al., 2011). Fis dissociation is believed to remove the steric barrier to IHF association, although the exact mechanism remains to be determined (Kaur et al., 2014; Leonard and Grimwade, 2015). The involvement of NAPs suggests the presence of long range interactions in the formation of nucleoprotein complexes at *oriC*. The importance of the relative distances between DnaA binding sites and their helical phasing is also suggestive of higher order structure formation (Woelker and Messer, 1993). Neither Fis nor IHF are essential *in vivo* or for replication *in vitro*; they are, however, required for regulating replication initiation in the cell cycle (Ryan et al., 2004). IHF can efficiently substitute for HU *in vitro*, indicating redundancy in NAPs requirement *in vivo* (Hwang and Kornberg, 1992a).

**Figure 3:**
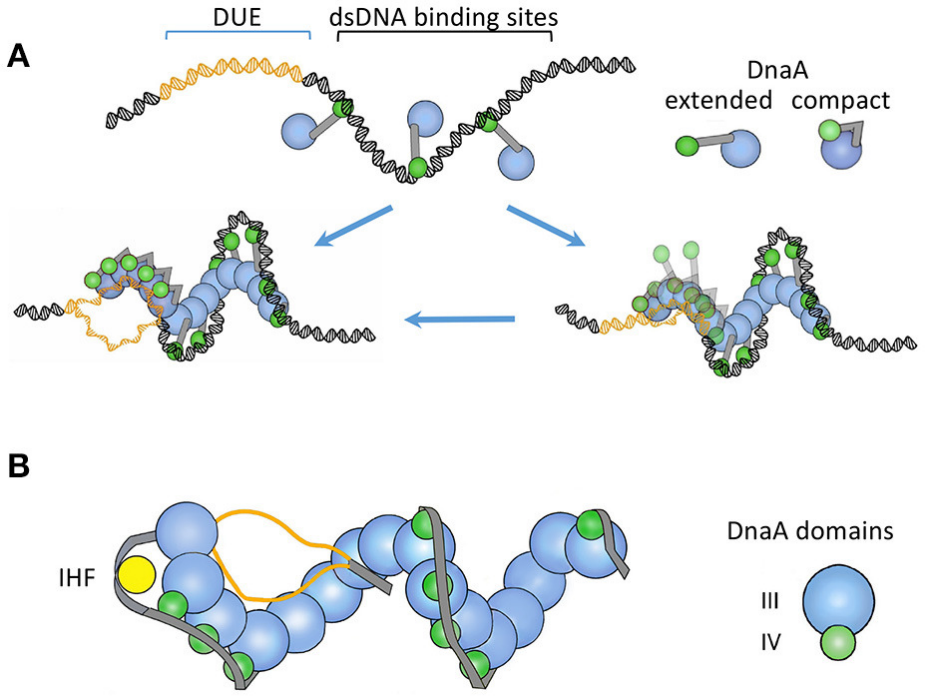
**(A)** A “two-state DnaA assembly model” for origin opening based primarily on crystallographic studies (Duderstadt et al., 2010). The figure is adapted from the authors' paper with permission from the publisher. The *oriC* regions where DnaA-ATP binds to DUE or to dsDNA are shown in different colors. In one state, the domain IV of DnaA stays extended and accessible for dsDNA binding. The binding initiates at the high-affinity sites and spreads to lower affinity sites with the increased availability of DnaA-ATP, as in Figure 2. Upon encountering the DUE, DnaA domain IV collapses on the AAA+ domain and becomes inaccessible for dsDNA binding. In this state, the AAA+ domain is used for ssDNA binding. The authors also considered the possibility that DnaA may initially bind to DUE when it is still ds (the right lower panel). **(B)** A “ssDUE recruitment model” based primarily on biochemical studies (Ozaki and Katayama, 2012). The figure is an adaptation from the authors' paper with permission from the publisher and shows DnaA without domains I and II. In this model, recruitment of one of the single strands of the DUE occurs by DnaA binding simultaneously to both DUE and dsDNA. The authors also considered that separate DnaA molecules bound to ss- or ds-DNA may interact with each other in DUE recruitment (not shown). The models in **(A)** and **(B)** both involve DnaA oligomerization and different DnaA domains for ss- and ds-DNA binding. The recruitment model incorporates additional features known to be important for opening: IHF binding, additional oligomerization through the N-terminal domain in organizing the open complex (not shown), and a spacer DNA between the DUE and R1 that is not bound by DnaA. The latter feature indicates that DnaA may not form a continuous spiral from dsDNA to the DUE as in **(A)**.

In addition to the eleven 9-mer sites, *oriC* contains three repeating 13-mer sequences that comprise the DUE, to which DnaA-ATP binds (Figures 1, 2; Speck and Messer, 2001). The 13-mers have adenine methylation sites, which, when methylated, are expected to favor strand separation (Gotoh and Tagashira, 1981). DnaA binding to DUE most likely requires the DUE to be single stranded, although initial binding may occur on dsDUE (Figure 3A; Duderstadt et al., 2011). The binding is mediated through the AAA+ domain of DnaA oligomers (Ozaki et al., 2008; Duderstadt et al., 2011). These details were obtained from X-ray crystallographic structures of N-terminal deleted DnaA from thermophilic bacteria (Erzberger et al., 2002; Ozaki et al., 2008). The DNA-protein and protein-protein contacts seen in crystals are also functionally significant in *E. coli* (Ozaki et al., 2008; Duderstadt et al., 2011).

Several important key findings have emerged from structural studies. Whereas DnaA-ADP is monomeric, DnaA-ATP is oligomeric (Erzberger et al., 2002, 2006; Ozaki et al., 2008; Ozaki and Katayama, 2012). The oligomerization is dependent on ATP, which bridges neighboring DnaA protomers at the interface between neighboring subunits by making contact with the Walker A and B motifs of one subunit and a conserved arginine (“arginine finger”) of the neighboring subunit through its γ-phosphate. The involvement of the γ-phosphate explains why DnaA-ADP fails to oligomerize. Mutating the arginine finger abolishes ATP-dependent binding of DnaA to *oriC* and initiation activity. Thus, oligomerization appears to be the mechanism to allow sequential binding to weak dsDNA sites and to the DUE (Cheng et al., 2015).

### Models for opening at *oriC*

DnaA-ATP in the crystal and in solution is a polymeric, right-handed spiral filament, which affords a ready explanation for how it could facilitate opening: Wrapping of DNA around a right-handed spiral is the same as introducing positive supercoils that could spontaneously induce compensatory negative supercoils in the adjoining DNA (Erzberger et al., 2006; Zorman et al., 2012). Although the negative supercoils can diffuse out of the origin, their proximity to wrapped DNA and propensity for melting render them more likely to be absorbed by unwinding of the DUE (Bowater et al., 1991; Polaczek et al., 1998). In this scenario, the wrapping of dsDNA around DnaA not only generates the unwinding force but also helps to confine the unwinding within the DUE. A stronger barrier to supercoil diffusion out of the A+T-rich DUE is suggested by the finding that the open state of DUE is quite stable in the absence of helicase loading *in vitro* and *in vivo* (Odegrip et al., 2000). Capturing one of the single strands by the AAA+ domain of DnaA oligomers as found in co-crystals could be a straightforward way to retain the DUE in the open state (Duderstadt et al., 2011).

DnaA may also directly open dsDUE (Figure 3A). This model is based on the structural similarities of ssDNA in complex with DnaA-ATP or RecA-ATP, and the biochemical evidence that DnaA can unwind short stretches of dsDNA (Duderstadt et al., 2011). RecA can transfer a single strand to homologous dsDNA (Shibata et al., 2001). Although cocrystals of DnaA-ATP with dsDNA are yet to be obtained, such structures were obtained with the archaeal initiator, Cdc6/Orc1, which is an AAA+ protein with significant homology to DnaA (Giraldo, 2003). The archaeal initiator was found to distort dsDNA, and DnaA also bends DNA upon binding (Schaper and Messer, 1995). Thus, similar to RecA, DnaA oligomers may initially contact dsDUE and distort the region enough to initiate ssDUE binding.

Structural studies indicate two distinct states of DnaA-ATP for ds- and ss-DNA binding. For contact with dsDNA, the C-terminal HTH domains of DnaA oligomers stick out of the spiral and are free to contact dsDNA as it wraps around the spiral from the outside (Figure 3A). For contact with ssDNA, the HTH domain collapses on the AAA+ domain of the partner protomer and can no longer contact dsDNA. The interaction between the collapsed HTH domain and the AAA+ domain is required for oligomerization-mediated ssDNA binding, origin opening, and initiation *in vivo* (Duderstadt et al., 2010). In other words, the HTH domain also contributes to DnaA oligomerization. What triggers the HTH domain to change its conformation from an extended to a collapsed state in DnaA oligomers is not understood. Another study suggested that ds- and ss-DNA binding can occur simultaneously on the same DnaA-ATP oligomer (Ozaki and Katayama, 2012; Figure 3B). When DUE sequences were provided as single-stranded oligos together with a DUE-deleted ds*oriC* fragment, the oligos could contact specific pore residues of the DnaA-ATP spiral. Mutating the contacting residues (V211A and R245A) prevented DUE binding and opening. Although it remains to be resolved whether the ds- and ss-DNA binding occur with separate or the same DnaA molecules, it is clear that DnaA oligomerization is important for origin opening and ssDNA binding. The importance of DnaA oligomerization has also been demonstrated in *Bacillus subtilis* (Scholefield et al., 2012).

Weak DnaA binding sites are clustered into two phased arrays that are oriented opposite to each other (Rozgaja et al., 2011; Figure 2B). The wrapping model appears inconsistent with this finding, because the handedness of wrapping is expected to be opposite for the two arrays and the torsional stress generated by wrapping of one array would be neutralized by wrapping of the other. However, the contribution of the two arrays to stress may not be equivalent. The DUE proximal array may be more important and can suffice for the opening. In fact, the deletion of the DUE distal array from *oriC* does not affect viability, and can achieve DUE opening, ssDNA binding and some DnaB loading *in vitro* (Stepankiw et al., 2009; Ozaki and Katayama, 2012). The DUE distal array becomes crucial during rapid growth and is required for enhancing helicase loading both *in vitro* and *in vivo* (Weigel et al., 2001; Stepankiw et al., 2009). Some elements of *oriC* might not be essential but they are there for improving its efficiency.

### Regulation of *oriC* opening

So far, we have discussed the importance of DnaA-ATP in regulating the opening, and the involvement of NAPs in this process. There are also other regulatory proteins that influence the opening by controlling DnaA interactions with *oriC*. Many of these regulators interact with the N-terminal domain of DnaA and modulate its oligomerization activity. Proteins HU and DiaA promote oligomerization and unwinding by DnaA (Hwang and Kornberg, 1992a; Chodavarapu et al., 2008a; Keyamura et al., 2009). There are also N-terminal domain binding proteins L2 and Dps that impede oligomerization and origin opening (Chodavarapu et al., 2008b, 2011). These N-terminal domain activities help in timing replication during the cell cycle and in maintaining replication synchrony during rapid growth, but are not essential for origin opening. Indeed, several studies have concluded that the essential role of the N–terminal domain is in the loading of the helicase (Sutton et al., 1998; Sharma et al., 2001; Speck and Messer, 2001; Simmons et al., 2003). However, DnaA cannot be loaded to low-affinity sites without an intact N-terminal domain, which would imply an essential role of the domain in opening (Miller et al., 2009). These apparent contradictions highlight the importance of clarifying the role of the N-terminal domain-mediated oligomerization.

There are also regulators that can indirectly control the opening of the DUE. Some regulators, such as SeqA and IciA, bind directly to the DUE and prevent opening by interfering with DnaA binding (Hwang and Kornberg, 1992b; Lu et al., 1994). SeqA also prevents DnaA binding to some of the low affinity sites that have overlapping SeqA binding sites (Nievera et al., 2006). Several other regulators control the DnaA-ATP level. These regulators have been reviewed comprehensively elsewhere, and will not be discussed here (Katayama et al., 2010; Skarstad and Katayama, 2013). Finally, for unknown reasons, transcription is required for replication initiation (Skarstad et al., 1990). The act of transcription elongation induces negative supercoiling of the upstream DNA. An appropriately oriented promoter may thus help origin opening by increasing negative supercoiling. This is further discussed below.

## Opening of the bacteriophage lambda origin by transcriptional activation

In phage lambda (λ), DNA replication has been extensively studied and is fairly well-understood. Before the days of cloning, the small size of the phage genome (about 50 kb, one 1/100th the size of the *E. coli* chromosome) made physical manipulation possible, allowing isolation and characterization of intact replication intermediates. This led to the first unambiguous demonstration that replication starts from a unique origin, and that two replication forks proceed from the origin in opposite directions (bidirectional replication), as was conceived in the replicon model (Jacob et al., 1964; Inman and Schnös, 1971).

Genetic characterization of lambda replication has provided quite a few alternate strategies for replication initiation. For example, instead of DnaA as the initiator, two phage-encoded initiators, the O and P proteins, are used (Ogawa and Tomizawa, 1968). The initiation depends on transcription within or nearby the origin region (Dove et al., 1969). A more remarkable finding was the discovery of three chaperone proteins in *E. coli* (DnaJ, DnaK, and GrpE) and their participation in replication initiation (Georgopoulos and Herskowitz, 1971; Saito and Uchida, 1977; Friedman et al., 1984). *In vitro* studies with purified components reproduced the salient features of the system as determined *in vivo*: bidirectional replication, and requirements for transcription and chaperone proteins (Learn et al., 1993). We elaborate on these features in the context of our general scheme of origin opening.

**Figure 4:**
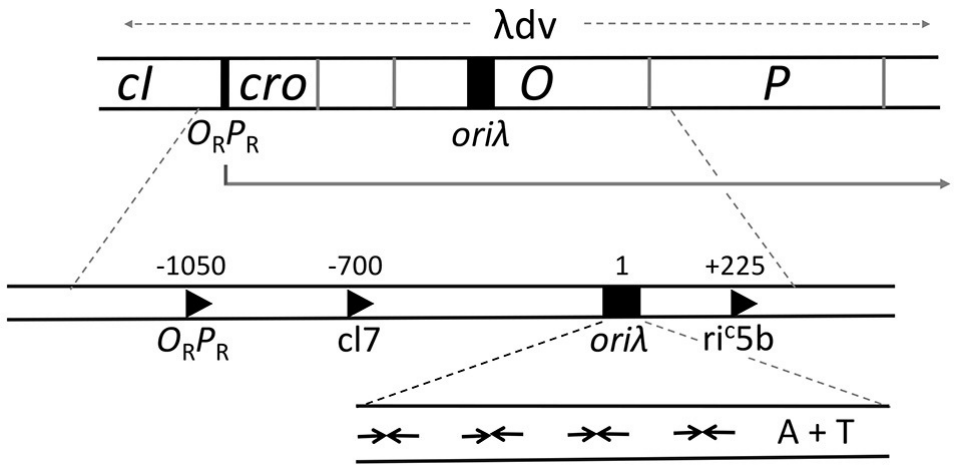
**Genetic map of the region required for λ replication**. A plasmid carrying the region marked λdv can replicate autonomously in *E. coli*. The region includes an operator/promoter (O_R_P_R_), which can be repressed by either cI or Cro protein. The three genes, *cro* and the initiators *O* and *P*, constitute an operon under O_R_P_R_ control. The origin, *ori*λ, maps within the *O* gene and requires activation by transcription from P_R_. When P_R_ is repressed, suppressor mutations that can activate the origin create new promoters (c17, ri^C^5b), which are not repressed by cI or Cro and are expressed constitutively. The expanded map of *ori*λ shows four inverted repeats (inverted arrows) for binding of O protein dimers followed by a 40 bp long A+T rich region.

The minimal region that retains the replication characteristics of the entire genome is contained within 2.4 kb (λdv, Figure 4). It comprises a promoter P_R_ and four genes *cro, cII, O*, and *P* that are transcribed from P_R_ (Matsubara, 1981). The origin (*ori*λ) maps within the *O* gene and transcription from P_R_ activates the origin, in addition to its role in providing the mRNA for O and P synthesis. *ori*λ contains nearly perfect inverted repeats of a 19-bp sequence that bind O protein dimers (Grosschedl and Hobom, 1979; Moore et al., 1979; Tsurimoto and Matsubara, 1981). The initiator binding repeats of the origin were given a special name, iterons (Moore et al., 1979). O binding to *ori*λ in a negatively supercoiled DNA causes a significant structural change that includes the opening of the neighboring 40 bp A+T rich region (Dodson et al., 1986; Schnos et al., 1988). The iteron DNA is bent in solution and bends further upon O binding, and it has been proposed that the “free energy of bending is trapped in the *ori*λ-O complex” (Zahn and Blattner, 1987). The opening is dependent on negative supercoiling and binding of O protein copies to each of the multiple iterons.

*In vitro* studies suggest that additional proteins are involved in stabilizing the opening to allow loading of the host-encoded helicase, DnaB. As in *oriC*, the initiation of λ replication is separated into several stages. The formation of the *ori*λ-O complex is the initial stage, followed by the formation of *ori*λ-O-P-DnaB, *ori*λ-O-P-DnaB-DnaJ and *ori*λ-O-P-DnaB-DnaJ-DnaK complexes (Alfano and McMacken, 1989a,b; Dodson et al., 1989). All these complexes are functional as they support replication when supplemented with the missing replisome components. In the *ori*λ-O-P-DnaB complex, P plays matchmaker by binding simultaneously to O and DnaB (Mallory et al., 1990; Osipiuk et al., 1993). DnaB is kept inactive at this stage through interaction with P, until chaperone proteins disassemble the complex to activate the helicase (Mensa-Wilmot et al., 1989b; Zylicz et al., 1989). The disassembly requires the ATPase activity of DnaK. The chaperones thus participate in initiation after the origin has opened.

O, P, and DnaB all harbor cryptic ssDNA binding activity (Learn et al., 1997). Interactions between O and P, and between P and DnaB, which suppress the intrinsic ssDNA binding activity of DnaB, are all required to form a stable ssDNA-O-P-DnaB complex. Both O and P of the complex contact the open DUE and stabilize the initial open structure. O also stabilizes the P-DnaB interaction, perhaps ensuring that DnaB is loaded only at the O-bound origin.

Although the chaperones provide a crucial activation function to the helicase, they do not control the efficiency of initiation or, most likely, strand-opening; these are controlled by transcription from the P_R_ (Thomas and Bertani, 1964; Dove et al., 1969). Hence, the repressors that control P_R_ activity are the regulators of replication. Within the minimal ~2.4 kb replicon (λdv), Cro serves as the repressor for P_R_, and in the intact phage, repression is enforced by the cI protein (Matsubara, 1981; Womble and Rownd, 1986). The two repressors bind to the same operator (O_R_) sequences. In the prophage state, when P_R_ is repressed by cI, replication does not initiate even when O and P are supplied *in trans* (Thomas and Bertani, 1964). Mutations that activate replication under the above conditions (Dove et al., 1969) were found to create new promoters that are not controlled by cI (e.g., c17, Figure 4). This observation led to the proposal that the λ origin requires activation by transcription. The transcription requirement has also been confirmed *in vitro* (Mensa-Wilmot et al., 1989a). In an RNA Polymerase dependent purified system, addition of cI abrogates replication initiation, but not in the presence of the c17 promoter. Later studies showed that the promoter could be downstream and directed away from the origin (e.g., ri^C^5b, Figure 4), implying that the origin region itself need not be transcribed (Furth et al., 1982).

The finding that new promoters located on either side of *ori*λ can activate the origin can be explained by the “twin supercoiled domain” model, where a transcribing RNA polymerase generates positive supercoils ahead of it and negative supercoils behind it (Liu and Wang, 1987). A common feature of the new promoters, regardless of whether they are ahead of or behind the origin, is that they are all oriented for rightward transcription, similar to P_R_. In other words, they are all disposed to increase negative superhelicity of the origin region; this is straightforward in the case of ri^C^5b which is downstream of *ori*λ, but when the promoter is upstream, as in the case of P_R_ or c17, transcription needs to proceed past the origin.

The requirement for transcriptional activation may be indirectly tied to increasing negative superhelicity. Notably, RNA polymerase is not required for *in vitro* λ replication with purified proteins (Mensa-Wilmot et al., 1989b). In the presence of HU, however, the purified system becomes dependent on RNA polymerase and transcription (Mensa-Wilmot et al., 1989a), which can sweep off HU from DNA. HU is known to constrain (reduce) negative supercoils, which could inhibit origin opening (Drlica and Rouviere-Yaniv, 1987; Mensa-Wilmot et al., 1989a). The superhelical density of plasmids isolated from cells appears adequate for replication *in vitro* but when it is reduced by HU binding, the role of transcription becomes obligatory. [Similarly, for *oriC*, transcription can activate replication under some conditions but is not required when purified proteins are used (Funnell et al., 1986)]. Transcription not only counters HU, but also makes replication initiation bidirectional (Learn et al., 1993). Without transcription, replication in the purified system almost always initiates unidirectionally, although *in vivo* it is primarily bidirectional (Schnos and Inman, 1970; Mensa-Wilmot et al., 1989b). How transcription significantly improves the frequency of bidirectional replication remains to be determined (Learn et al., 1993). Transcription and negative supercoiling may also contribute in additional ways (Szambowska et al., 2011). The RNA polymerase β subunit makes a direct contact with the O protein, and this interaction is stimulated by negative supercoiling. Thus, lowering the energy required for DNA strand separation may not be the only role of negative supercoiling.

## Opening of origins in plasmids with repeated initiator binding sites (iterons)

**Figure 5:**
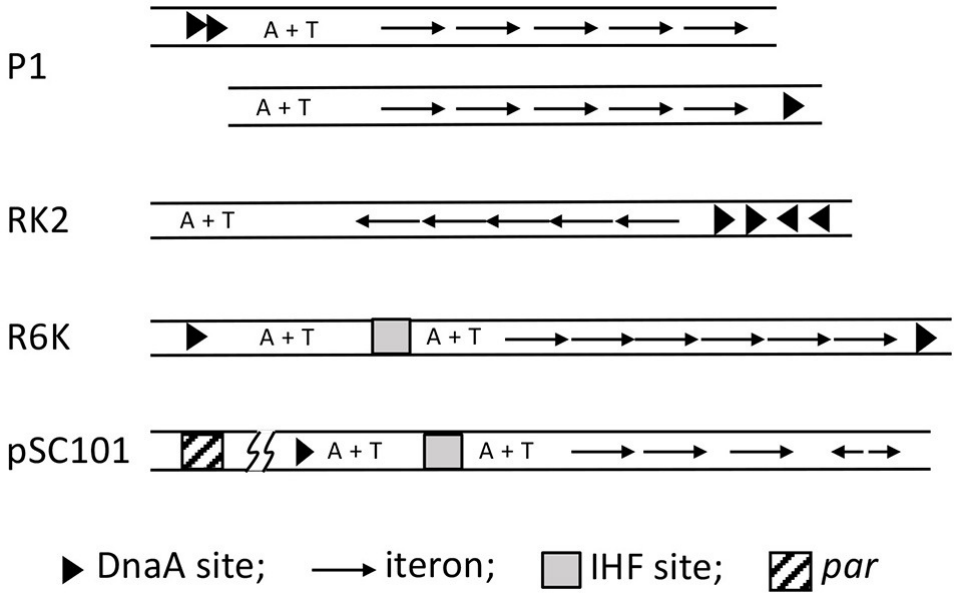
**Maps of iteron-bearing plasmids**. The plasmid iterons are mostly direct repeats, as opposed to the inverted repeats in *ori*λ. Two P1 maps are shown, the top one being the wild type and the one below with the DnaA binding sites (arrow heads) deleted and one of them moved next to the iterons. Both of the origins are functional, indicating that the A+T rich region does not have to be bounded by protein binding sites. In the RK2 plasmid, the A+T rich region naturally lacks protein binding sites in one of its flanks. By contrast, the R6K *ori*γ is bounded by DnaA sites, which most likely interact directly. pSC101 uniquely includes a *par* locus (about 200 bp away from the origin as indicated by the line breaks), which binds gyrase and specifically changes the negative superhelicity of the origin, and thereby enhances replication of the plasmid.

The basic feature of the lambda origin, namely, an array of repeats of replicon-specific initiator binding sites (iterons), can be found in the origins of a large family of bacterial plasmids (Figure 5; Chattoraj and Schneider, 1997). Unlike λ iterons, which bind O dimers, plasmid iterons bind monomeric initiators, a feature that is important for regulating replication, as discussed later. Plasmid iterons are generally present in phase with the helical repeat of B-DNA, and disturbing the phasing can inactivate the origin (Brendler et al., 1997; Doran et al., 1998). The presence of phased iterons indicates that the plasmid origins assume a higher order structure, as appears to be the case for *oriC* and *ori*λ.

Apart from iterons, the plasmid origins have binding sites for DnaA and a NAP, both of which are required for the origin function. However, the AAA+ (ATPase) domain of DnaA is not required for plasmid replication, suggesting DnaA plays a less crucial role in plasmid replication than in chromosomal replication (Lu et al., 1998; Sharma et al., 2001). Plasmid replication is controlled instead by dimerization of plasmid specific initiators (Paulsson and Chattoraj, 2006). Chaperones are involved in plasmid replication, but unlike their role in λ replication, they control the dimerization efficiency of the initiator and are not involved in activation of the replicative helicase (Wickner et al., 1991). In spite of these differences, the origin opening mechanism is believed to follow the λ paradigm, namely, distortion of the origin by initiator binding to the iteron array with cooperation from DnaA and NAP binding, resulting in opening the A+T rich region. However, unlike λ replication, transcription is not known to be a requirement for *in vivo* plasmid replication.

Origin opening has been studied in several of the iteron-based plasmids, including P1 (Mukhopadhyay et al., 1993), F (Kawasaki et al., 1996), RK2 (Konieczny et al., 1997), pSC101 (Sharma et al., 2001), and R6K (Lu et al., 1998; Krüger et al., 2001). The roles of the plasmid initiator (usually called Rep), DnaA and NAP vary depending upon the plasmid. In plasmid P1, DnaA alone can initiate opening, but it is greatly facilitated by the addition of RepA (Mukhopadhyay et al., 1993). RepA alone is ineffective. In plasmid F also DnaA alone can open the origin but neither the initiator RepE nor the NAP (HU) alone can do so (Kawasaki et al., 1996). Together, RepE and HU are efficient in opening. Addition of DnaA further increases the efficiency of opening and extends the open region. In plasmid RK2, Rep (TrfA) can open if either HU or DnaA is present (Konieczny et al., 1997). Opening by TrfA together with HU is significantly improved when DnaA is also present. In pSC101, cooperation of DnaA, RepA, and IHF is required to open the origin efficiently (Sharma et al., 2001). All three, Rep (pi), DnaA and a NAP (IHF) are also required to open *ori*γ of R6K (Krüger et al., 2001; Lu et al., 1998). However, with a hyperactive variant of pi, DnaA and IHF are not required, indicating that their roles are mostly facilitatory (Krüger and Filutowicz, 2003). The general picture that emerges is that although some opening might be seen without the full complement of the three proteins, the efficiency and/or the extent of the opening are usually different in such cases.

The above studies indicate a direct correlation between the efficiency of origin opening and replication initiation. In P1 and F, situations that increase or decrease initiation due to changes in Rep or iteron concentration also correspondingly enhance or reduce opening (Kawasaki et al., 1996; Park et al., 1998; Park and Chattoraj, 2001; Zzaman and Bastia, 2005). In RK2, whose DUE comprises 13-mer A+T rich repeats like the DUE of *oriC*, changing their sequence, arrangement, or number reduces the stability of the open DUE as well as the origin firing efficiency (Rajewska et al., 2008; Wegrzyn et al., 2014). In pSC101, a RepA mutant specifically defective in interactions with DnaA and replication initiation is also defective in origin opening (Sharma et al., 2001). In R6K, both the monomer and dimer forms of pi bind and bend iterons almost equally, but only the monomer-bound origins can open, which is the form that is proficient in initiation (Krüger et al., 2001; Krüger and Filutowicz, 2003). As mentioned earlier, the facilitators of opening of *ori*γ, IHF and DnaA, are also required for initiation. The pi mutants that can open without the facilitators are also hyperactive (copy-up) for initiation. These results suggest that initiators control initiation efficiency at the DNA-opening step (Krüger et al., 2001).

In plasmids RK2 and F, the initiators bind ssDUE, as we have described for DnaA binding to *oriC*-DUE, and O and P binding to *ori*λ-DUE (Wegrzyn et al., 2014). In iteron-bearing plasmids, similar to *oriC* or *ori*λ, the A+T region is not always flanked by protein binding sites that might prevent migration of the opening away from the origin. Even in cases where the DUE is flanked by DnaA and RepA binding sites, the DnaA binding sites can be moved to the other end of the origin, so that the plasmid origin now mimics *oriC* or *ori*λ (P1 origin, Figure 5; Abeles et al., 1990; Park and Chattoraj, 2001). These results argue in favor of active anchoring mechanisms. Indeed in RK2 and F, iteron-bound initiators can bind simultaneously to an oligo from DUE, as they can at *oriC* (Ozaki and Katayama, 2012; Wegrzyn et al., 2014). While DnaA uses two different domains for binding to ds- and ss-DNA, it is not known whether that is also the case for plasmid initiators.

In many iteron-based plasmids, chaperones improve initiator–iteron binding that leads to opening. This is in contrast to their roles in λ, where the chaperones come into play after origin opening. In plasmids, the chaperones increase the availability of initiator monomers in a form that binds to iterons (Wickner et al., 1991; Ishiai et al., 1994; Toukdarian et al., 1996; Zzaman et al., 2004b). The increase in monomer results from dimer dissociation *in vitro* and this results from refolding of misfolded subunits that apparently reduces dimerization affinity (Giraldo et al., 2003; Nakamura et al., 2007). The chaperones could be either DnaK and its cohorts DnaJ and GrpE for P1 RepA (Wickner et al., 1991), F RepE (Ishiai et al., 1994) and R6K pi (Zzaman et al., 2004b), or only ClpA for P1 RepA (Wickner et al., 1994), or ClpB+DnaK+DnaJ+GrpE as for RK2 TrfA (Konieczny and Liberek, 2002). Although replication of these plasmids require the monomers, prevention of over-replication requires the dimers (Paulsson and Chattoraj, 2006). The chaperones thus play an important role in maintaining the proper balance between the activator (monomer) and inhibitor (dimer) forms of the initiators.

Origins of iteron-based plasmids are generally not dependent on transcription. As in *E. coli* and λ replication, RNA polymerase is not required for R6K and F replication when purified components are used (Abhyankar et al., 2003; Zzaman et al., 2004a). However, as in other systems, RNA polymerase has been shown to play a beneficial role in pSC101 replication *in vivo*. The plasmid has a locus, *par*, for gyrase binding that increases negative supercoiling specifically at the plasmid origin (Miller et al., 1990; Conley and Cohen, 1995). The supercoiling and replication defects of Δ*par* strains are suppressed by transcription from a suitably positioned and oriented promoter (Beaucage et al., 1991). These results are consistent with the “twin supercoiled domain” model but also support the view that superhelicity can be changed locally without changing the overall superhelical density of the plasmid (Rahmouni and Wells, 1989). The localized changes are believed to improve initiator interactions with the origin and thereby its activity (Ingmer and Cohen, 1993).

## Opening of colE1 plasmid origin by formation of a persistent RNA-DNA hybrid

**Figure 6:**
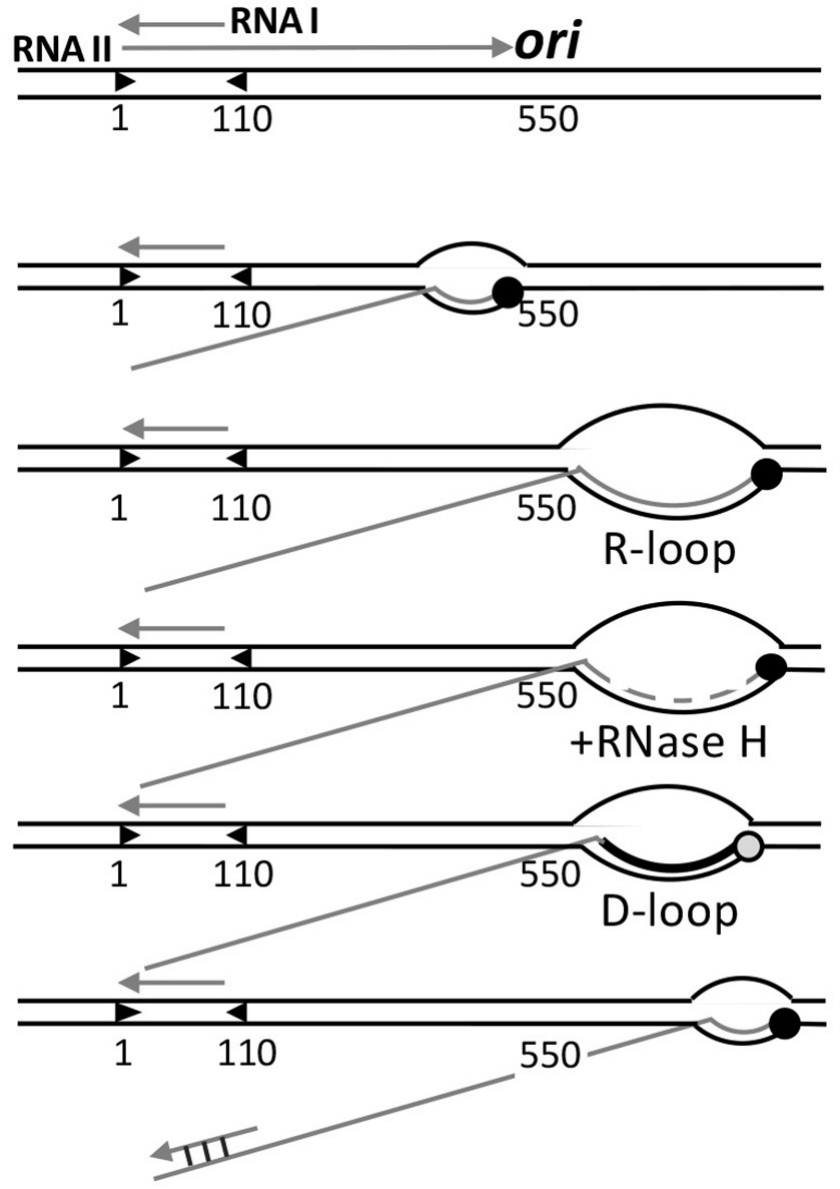
**Control of origin opening in plasmid ColE1**. Two transcripts (RNA I and RNAII) control opening of the origin. RNA II initiates and elongates normally up to 550 nt, where it starts to form a persistent hybrid that increases the size of R-loop from a normal size of 10 nt to more than 200 nt. The hybridized RNA is degraded all but a few nt by RNase H, and this residual hybridized RNA serves as the primer for DNA synthesis by Pol I, which converts the unstable R-loop to a stable D-loop. The non-template strand of the D-loop is then used for helicase loading. The hybridization of RNA I with the 5′-end of RNA II (covering nt 2–110) negatively regulates replication by changing the secondary structure of RNA II that thwarts persistent R-loop formation, without which DNA synthesis is not primed.

Replication initiation of plasmid ColE1 differs from that of the replicons described above. ColE1 does not use a plasmid-encoded initiator. Rather, initiation depends on the host RNA polymerase, which synthesizes a non-coding RNA (RNA II) from a promoter 550 bp upstream of the origin. This serves to open the origin and provides the primer for DNA synthesis (Figure 6). This diverges from the norm for bacterial replicons, where the primer is synthesized by the primase, DnaG, which is brought to the open origin by DnaB-DnaG protein-protein interactions (Bell and Kaguni, 2013).

In transcription elongation, normally about 10 nt at the 3′-end of transcripts stay hybridized to the template strand, forming a three-stranded bubble called the R-loop. As new nucleotides are added at the 3′-end, the hybridized nucleotides at the 5′-end of R-loops leave the template strand, thereby maintaining the size of the translocating R-loops. This canonical scenario is maintained in the case of RNA II for the first 550 nt, after which the RNA does not exit from the R-loop as new nucleotides are added. The persistence of hybridized RNA causes the R-loop to grow in size to even more than 200 bp. [Persistence of RNA-DNA hybrid was also found in transcriptional activation of *oriC* (Baker and Kornberg, 1988)]. RNase H then almost completely degrades the RNA from the R-loop except for the 4 to 5 hybridized nucleotides at the 5′-end side. The residual hybridized RNA suffices to serve as the primer to start DNA synthesis by DNA polymerase I (Pol I). *In vivo*, RNase H most likely prevents the R-loops to expand much in length, which needs to be at least 40 nt to allow helicase loading (Masukata et al., 1987). The three stranded D-loop synthesized by Pol I apparently provides sufficient opening for the helicase. The open (D-loop) region does not have to be A+T rich in this case because the opening is caused by DNA synthesis, not from the intrinsic instability of the region. The D-loop is a stable structure wherein the newly synthesized strand prevents the non-template strand from hybridizing back to the template strand.

Initiation is controlled by the prevention of persistent RNA-DNA hybridization. A shorter RNA (RNA I) about 110 nt long and fully complementary to the 5′-end of RNA II is responsible for preventing R-loop expansion. RNA I and RNAII are both constitutively synthesized. As their concentrations increase with increasing plasmid copy number, hybridization becomes increasingly significant. Hybridization changes the secondary structure of RNA II that thwarts persistent R-loops formation, and hence, priming.

In sum, although the initiation of ColE1 replication is mechanistically distinct, it espouses the two features highlighted here: the need for stabilizing the open state and the use of the origin opening stage to control initiation. It should be noted that in ColE1, transcription plays a direct and essential role in initiation by providing the primer, whereas in other cases transcription helps indirectly by increasing mainly negative superhelicity and is not obligatory. Finally, the study of ColE1 replication provided the first example of control by a non-coding antisense RNA, which is now recognized to be widespread in biology (Tomizawa et al., 1981; Eguchi et al., 1991).

## Origin opening in eukaryotes

The bacterial program of first opening the origin and then loading two hexameric helicases sequentially for bidirectional replication is not conserved in archaea and eukaryotes. In the latter, both the helicases are loaded together as a double hexamer to an unopened ds–origin (Bell and Kaguni, 2013). The loading otherwise follows the basic bacterial paradigm: the helicase (a hetero-hexamer, MCM2-7) in association with the helicase loader Cdt1, is recruited to the origin bound by the initiator (ORC) as a complex with another factor called Cdc6. The double hexamer is loaded in the post-mitotic, early G1 phases of the cell cycle in an inactive state and as a ring that encircles the ds-origin in its central core. The helicase activation and strand separation occur later in S-phase where the double hexamer is converted to single hexameric rings, each encircling one single strand for bidirectional movement. These major transitions require S-phase kinases and several additional factors, the details of which are under current investigation (Yardimci and Walter, 2014; Bochman and Schwacha, 2015; Petojevic et al., 2015). Loading and activation of the helicase at different stages of the cell cycle help to restrict initiation to only once per cell cycle (Nguyen et al., 2001; Arias and Walter, 2007). Since no new helicases can be loaded in S-phase, new origin firing cannot happen either. Thus, although the mechanisms of helicase loading have been largely conserved, the mechanisms of helicase activation and origin opening have diverged in different domains of life.

## Conclusions and future considerations

Here we have provided a few examples of how bacterial origins open, permitting loading of the replicative helicase. As some opening is possible without initiators, it is likely that the origin is inherently unstable (Gille and Messer, 1991; Mukhopadhyay and Chattoraj, 1993; Polaczek et al., 1998). Initiator binding pushes the propensity of opening over the threshold. Of all the requirements for opening, the free energy of negative supercoiling of the origin region appears to be the most basic requirement (Miller et al., 1990). Many of the facilitators of opening (e.g., transcription, NAPs, and pSC101*par*) work through changing superhelicity of the origin region. The activated state of supercoiled DNA facilitates changes of the DNA structure, events that are less likely to occur with linear DNA.

Initiator multimerization appears to be a general contributor to origin opening. This can allow wrapping of DNA with initiator and consequently changing the superhelicity of neighboring DNA. DNA binding proteins usually bend the DNA, and the initiators are no exception (Mukherjee et al., 1985; Mukhopadhyay and Chattoraj, 1993; Schaper and Messer, 1995). Stress from DNA bending can induce base-pair opening (Kahn et al., 1994). [The NAP binding can also untwist DNA (Teter et al., 2000)]. The opening by bending is initially local but the unwinding may migrate. Reducing the number of initiator binding sites in DNA generally makes the origin inefficient or inactive, depending upon the degree of binding site reduction.

Multimerization is also involved in ssDNA binding, which either stabilizes the open region or promotes actual unwinding (or both; Figure 3A). What triggers the conformational switch in initiators that allows them to bind ssDNA remains a challenging question (Duderstadt et al., 2011). More structural studies of the complexes are in order to get further insights into the opening process. This is now a realizable goal given the recent progress in cryo-EM (Merk et al., 2016). Even in the relatively clear case of ColE1, the trigger that causes the RNA polymerase to start forming persistent RNA-DNA hybrids when it encounters the origin sequence remains speculative.

Although we have referred to the DUE simply as A+T rich, the exact sequence of the region also matters (Hwang and Kornberg, 1992b; Ozaki et al., 2008). In RK2, the A+T rich region has 13-mers that bear partial homology to the *E. coli* 13-mers, but they are not interchangeable (Kowalczyk et al., 2005). In λ, the sequence of the A+T rich region is highly strand asymmetric: almost all purines are in one strand (Schnos et al., 1988). Paradoxically, such extreme asymmetric distribution of purine and pyrimidines stiffens the DNA more than DNA with more random sequences (Wells et al., 1970). In most cases, a specific strand is captured in the open region (Mukhopadhyay et al., 1993; Rajewska et al., 2008; Wegrzyn et al., 2014). Strand capture preference is also observed in experiments where the single strands are supplied *in trans* (Ozaki et al., 2008; Rajewska et al., 2008; Wegrzyn et al., 2014). A recent study has revealed a repeating trinucleotide motif that is conserved in bacterial DUEs and is required for origin opening (Richardson et al., 2016).

A recent study has provided a new insight into how supercoiling-induced DNA opening of A+T rich sequences is favored by G+C rich flanks (Vlijm et al., 2015). Such flanks are found in A+T rich stretches of some replication origins but their role has remained unclear (Brendler et al., 1991; Richardson et al., 2016). G+C rich stretches were suggested to resist transmission of torsional stress along the DNA (Skarstad et al., 1990). The recent study suggests further that the G+C stretches because of their stiffness resist (plectoneme formation) supercoiling of under-wound DNA and thereby help to concentrate the unwinding to A+T rich regions.

ATP not only plays an essential role in origin opening by AAA+ initiators, it also plays a rather mysterious role in opening origins where the initiators have no known ATP binding domain or ATPase activity. For example, pi binding and bending are not sufficient to open R6K *ori*γ without the presence of ATP (Krüger and Filutowicz, 2003). ATP, although not required, enlarges opening in RK2 even when a hyperactive mutant TrfA is used in conjunction with the facilitators DnaA and HU (Konieczny et al., 1997). Also at *oriC*, ATP requirements for DNA binding and origin opening by DnaA can vary by orders of magnitude (nM vs. mM; Figure 1; Bramhill and Kornberg, 1988a). A high ATP concentration can cause a conformation change in DnaA that appears likely to be required for opening (Saxena et al., 2015).

In closing, we prefer the view that the opening proceeds in steps rather than by a highly cooperative transition (the two models in Figure 3A). Initiator binding initiates the opening and it is further enhanced by multimerization of the initiator, facilitators like DnaA and NAPs (in plasmids) and by factors such as the helicase loaders DnaC and λP that have ssDNA binding activity. The opening by initiators alone may not be sufficient for helicase loading. The involvement of multiple factors provides multiple opportunities for regulation.

## Author contributions

All authors listed, have made substantial, direct and intellectual contribution to the work, and approved it for publication.

## Funding

Our funding is from the Intramural Research Program of the Center for Cancer Research, NCI, NIH.

### Conflict of interest statement

The authors declare that the research was conducted in the absence of any commercial or financial relationships that could be construed as a potential conflict of interest.

## References

[B1] Abeles A. L. Reaves L. D. Austin S. J. (1990). A single DnaA box is sufficient for initiation from the P1 plasmid origin. J. Bacteriol. 172, 4386–4391. 2165477 10.1128/jb.172.8.4386-4391.1990 PMC213265

[B2] Abhyankar M. M. Zzaman S. Bastia D. (2003). Reconstitution of R6K DNA replication *in vitro* using 22 purified proteins. J. Biol. Chem. 278, 45476–45484. 10.1074/jbc.M308516200 12970346

[B3] Alfano C. McMacken R. (1989a). Heat shock protein-mediated disassembly of nucleoprotein structures is required for the initiation of bacteriophage lambda DNA replication. J. Biol. Chem. 264, 10709–10718. 2543679

[B4] Alfano C. McMacken R. (1989b). Ordered assembly of nucleoprotein structures at the bacteriophage lambda replication origin during the initiation of DNA replication. J. Biol. Chem. 264, 10699–10708. 2525129

[B5] Arias E. E. Walter J. C. (2007). Strength in numbers: preventing rereplication via multiple mechanisms in eukaryotic cells. Genes Dev. 21, 497–518. 10.1101/gad.1508907 17344412

[B6] Baker T. A. Kornberg A. (1988). Transcriptional activation of initiation of replication from the *E. coli* chromosomal origin: an RNA-DNA hybrid near oriC. Cell 55, 113–123. 10.1016/0092-8674(88)90014-1 2458841

[B7] Beaucage S. L. Miller C. A. Cohen S. N. (1991). Gyrase-dependent stabilization of pSC101 plasmid inheritance by transcriptionally active promoters. EMBO J. 10, 2583–2588. 1651231 10.1002/j.1460-2075.1991.tb07799.x PMC452956

[B8] Bell S. P. Kaguni J. M. (2013). Helicase loading at chromosomal origins of replication. Cold Spring Harb. Perspect. Biol. 5:a010124. 10.1101/cshperspect.a010124 23613349 PMC3660832

[B9] Bochman M. L. Schwacha A. (2015). DNA replication: strand separation unravelled. Nature 524, 166–167. 10.1038/nature14643 26222029

[B10] Bowater R. Aboul-ela F. Lilley D. M. (1991). Large-scale stable opening of supercoiled DNA in response to temperature and supercoiling in (A + T)-rich regions that promote low-salt cruciform extrusion. Biochemistry 30, 11495–11506. 10.1021/bi00113a003 1747368

[B11] Bramhill D. Kornberg A. (1988a). Duplex opening by DnaA protein at novel sequences in initiation of replication at the origin of the *E. coli* chromosome. Cell 52, 743–755. 2830993 10.1016/0092-8674(88)90412-6

[B12] Bramhill D. Kornberg A. (1988b). A model for initiation at origins of DNA replication. Cell 54, 915–918. 2843291 10.1016/0092-8674(88)90102-x

[B13] Brendler T. Abeles A. Austin S. (1991). Critical sequences in the core of the P1 plasmid replication origin. J. Bacteriol. 173, 3935–3942. 2061278 10.1128/jb.173.13.3935-3942.1991 PMC208038

[B14] Brendler T. G. Abeles A. L. Reaves L. D. Austin S. J. (1997). The iteron bases and spacers of the P1 replication origin contain information that specifies the formation of a complex structure involved in initiation. Mol. Microbiol. 23, 559–567. 10.1046/j.1365-2958.1997.d01-1869.x 9044289

[B15] Chattoraj D. K. Schneider T. D. (1997). Replication control of plasmid P1 and its host chromosome: the common ground. Prog. Nucleic Acid Res. Mol. Biol. 57, 145–186. 10.1016/S0079-6603(08)60280-9 9175433

[B16] Cheng H. M. Groger P. Hartmann A. Schlierf M. (2015). Bacterial initiators form dynamic filaments on single-stranded DNA monomer by monomer. Nucleic Acids Res. 43, 396–405. 10.1093/nar/gku1284 25477384 PMC4288177

[B17] Chodavarapu S. Felczak M. M. Kaguni J. M. (2011). Two forms of ribosomal protein L2 of *Escherichia coli* that inhibit DnaA in DNA replication. Nucleic Acids Res. 39, 4180–4191. 10.1093/nar/gkq1203 21288885 PMC3105425

[B18] Chodavarapu S. Felczak M. M. Yaniv J. R. Kaguni J. M. (2008a). *Escherichia coli* DnaA interacts with HU in initiation at the *E. coli* replication origin. Mol. Microbiol. 67, 781–792. 10.1111/j.1365-2958.2007.06094.x 18179598

[B19] Chodavarapu S. Gomez R. Vicente M. Kaguni J. M. (2008b). *Escherichia coli* Dps interacts with DnaA protein to impede initiation: a model of adaptive mutation. Mol. Microbiol. 67, 1331–1346. 10.1111/j.1365-2958.2008.06127.x 18284581

[B20] Conley D. L. Cohen S. N. (1995). Effects of the pSC101 partition (*par*) locus on *in vivo* DNA supercoiling near the plasmid replication origin. Nucleic Acids Res. 23, 701–707. 10.1093/nar/23.4.701 7899092 PMC306741

[B21] Dodson M. Echols H. Wickner S. Alfano C. Mensa-Wilmot K. Gomes B.. (1986). Specialized nucleoprotein structures at the origin of replication of bacteriophage lambda: localized unwinding of duplex DNA by a six-protein reaction. Proc. Natl. Acad. Sci. U.S.A. 83, 7638–7642. 10.1073/pnas.83.20.7638 3020552 PMC386776

[B22] Dodson M. McMacken R. Echols H. (1989). Specialized nucleoprotein structures at the origin of replication of bacteriophage lambda. Protein association and disassociation reactions responsible for localized initiation of replication. J. Biol. Chem. 264, 10719–10725. 2525130

[B23] Doran K. S. Konieczny I. Helinski D. R. (1998). Replication origin of the broad host range plasmid RK2. Positioning of various motifs is critical for initiation of replication. J. Biol. Chem. 273, 8447–8453. 10.1074/jbc.273.14.8447 9525957

[B24] Dorman C. J. (2009). Nucleoid-associated proteins and bacterial physiology. Adv. Appl. Microbiol. 67, 47–64. 10.1016/S0065-2164(08)01002-2 19245936

[B25] Dove W. F. Hargrove E. Ohashi M. Haugli F. Guha A. (1969). Replicator activation in lambda. Japan J Genet 44, 11–22.

[B26] Drlica K. Rouviere-Yaniv J. (1987). Histonelike proteins of bacteria. Microbiol. Rev. 51, 301–319. 3118156 10.1128/mr.51.3.301-319.1987 PMC373113

[B27] Duderstadt K. E. Chuang K. Berger J. M. (2011). DNA stretching by bacterial initiators promotes replication origin opening. Nature 478, 209–213. 10.1038/nature10455 21964332 PMC3192921

[B28] Duderstadt K. E. Mott M. L. Crisona N. J. Chuang K. Yang H. Berger J. M. (2010). Origin remodeling and opening in bacteria rely on distinct assembly states of the DnaA initiator. J. Biol. Chem. 285, 28229–28239. 10.1074/jbc.M110.147975 20595381 PMC2934688

[B29] Eguchi Y. Itoh T. Tomizawa J. (1991). Antisense RNA. Annu. Rev. Biochem. 60, 631–652. 10.1146/annurev.bi.60.070191.003215 1715680

[B30] Erzberger J. P. Mott M. L. Berger J. M. (2006). Structural basis for ATP-dependent DnaA assembly and replication-origin remodeling. Nat. Struct. Mol. Biol. 13, 676–683. 10.1038/nsmb1115 16829961

[B31] Erzberger J. P. Pirruccello M. M. Berger J. M. (2002). The structure of bacterial DnaA: implications for general mechanisms underlying DNA replication initiation. EMBO J. 21, 4763–4773. 10.1093/emboj/cdf496 12234917 PMC126292

[B32] Friedman D. I. Olson E. R. Georgopoulos C. Tilly K. Herskowitz I. Banuett F. (1984). Interactions of bacteriophage and host macromolecules in the growth of bacteriophage lambda. Microbiol. Rev. 48, 299–325. 6240590 10.1128/mr.48.4.299-325.1984 PMC373221

[B33] Funnell B. E. Baker T. A. Kornberg A. (1986). Complete enzymatic replication of plasmids containing the origin of the *Escherichia coli* chromosome. J. Biol. Chem. 261, 5616–5624. 3514619

[B34] Furth M. E. Dove W. F. Meyer B. J. (1982). Specificity determinants for bacteriophage lambda DNA replication. III. Activation of replication in lambda ri^*c*^ mutants by transcription outside of *ori*. J. Mol. Biol. 154, 65–83. 10.1016/0022-2836(82)90417-X 6210781

[B35] Georgopoulos C. Herskowitz I. (1971). *Escherichia coli* mutants blocked in lambda DNA synthesis, in The Bacteriophage Lambda, ed Hershey A. D. (New York, NY: Cold Spring Harbor laboratory), 553–564.

[B36] Gille H. Messer W. (1991). Localized DNA melting and structural pertubations in the origin of replication, *oriC*, of *Escherichia coli in vitro* and *in vivo*. EMBO J. 10, 1579–1584. 2026151 10.1002/j.1460-2075.1991.tb07678.x PMC452823

[B37] Giraldo R. (2003). Common domains in the initiators of DNA replication in Bacteria, Archaea and Eukarya: combined structural, functional and phylogenetic perspectives. FEMS Microbiol. Rev. 26, 533–554. 10.1111/j.1574-6976.2003.tb00629.x 12586394

[B38] Giraldo R. Fernández-Tornero C. Evans P. R. Díaz-Orejas R. Romero A. (2003). A conformational switch between transcriptional repression and replication initiation in the RepA dimerization domain. Nat. Struct. Biol. 10, 565–571. 10.1038/nsb937 12766757

[B39] Gotoh O. Tagashira Y. (1981). Locations of frequently opening regions on natural DNAs and their relation to functional loci. Biopolymers 20, 1043–1058. 10.1002/bip.1981.360200514 7225530

[B40] Grosschedl R. Hobom G. (1979). DNA sequences and structural homologies of the replication origins of lambdoid bacteriophages. Nature 277, 621–627. 10.1038/277621a0 423961

[B41] Hwang D. S. Kornberg A. (1992a). Opening of the replication origin of *Escherichia coli* by DnaA protein with protein HU or IHF. J. Biol. Chem. 267, 23083–23086. 1429655

[B42] Hwang D. S. Kornberg A. (1992b). Opposed actions of regulatory proteins, DnaA and IciA, in opening the replication origin of *Escherichia coli*. J. Biol. Chem. 267, 23087–23091. 1429656

[B43] Ingmer H. Cohen S. N. (1993). The pSC101 *par* locus alters protein-DNA interactions *in vivo* at the plasmid replication origin. J. Bacteriol. 175, 6046–6048. 8376350 10.1128/jb.175.18.6046-6048.1993 PMC206688

[B44] Inman R. B. (1966). A denaturation map of the lambda phage DNA molecule determined by electron microscopy. J. Mol. Biol. 18, 464–476. 10.1016/S0022-2836(66)80037-2 5966296

[B45] Inman R. B. Schnös M. (1971). Structure of branch points in replicating DNA: presence of single-stranded connections in lambda DNA branch points. J. Mol. Biol. 56, 319–325. 10.1016/0022-2836(71)90467-0 4927949

[B46] Ishiai M. Wada C. Kawasaki Y. Yura T. (1994). Replication initiator protein RepE of mini-F plasmid: functional differentiation between monomers (initiator) and dimers (autogenous repressor). Proc. Natl. Acad. Sci. U.S.A. 91, 3839–3843. 10.1073/pnas.91.9.3839 8170998 PMC43677

[B47] Jacob F. Brenner S. Cuzin F. (1964). On the regulation of DNA replication in bacteria. *Cold Spring Harb*. Symp. Quant. Biol. 28, 329–348. 10.1101/SQB.1963.028.01.048

[B48] Kaguni J. M. Kornberg A. (1984). Replication initiated at the origin (*oriC*) of the *E. coli* chromosome reconstituted with purified enzymes. Cell 38, 183–190. 10.1016/0092-8674(84)90539-7 6088063

[B49] Kahn J. D. Yun E. Crothers D. M. (1994). Detection of localized DNA flexibility. Nature 368, 163–166. 10.1038/368163a0 8139661

[B50] Katayama T. Ozaki S. Keyamura K. Fujimitsu K. (2010). Regulation of the replication cycle: conserved and diverse regulatory systems for DnaA and *oriC*. Nat. Rev. Microbiol. 8, 163–170. 10.1038/nrmicro2314 20157337

[B51] Kaur G. Vora M. P. Czerwonka C. A. Rozgaja T. A. Grimwade J. E. Leonard A. C. (2014). Building the bacterial orisome: high affinity DnaA recognition plays a role in setting the conformation of *oriC* DNA. Mol. Microbiol. 91, 1148–1163. 10.1111/mmi.12525 24443848 PMC3992943

[B52] Kawakami H. Keyamura K. Katayama T. (2005). Formation of an ATP-DnaA-specific initiation complex requires DnaA Arginine 285, a conserved motif in the AAA+ protein family. J. Biol. Chem. 280, 27420–27430. 10.1074/jbc.M502764200 15901724

[B53] Kawasaki Y. Matsunaga F. Kano Y. Yura T. Wada C. (1996). The localized melting of mini-F origin by the combined action of the mini-F initiator protein (RepE) and HU and DnaA of *Escherichia coli*. Mol. Gen. Genet. 253, 42–49. 10.1007/s004380050294 9003285

[B54] Keyamura K. Abe Y. Higashi M. Ueda T. Katayama T. (2009). DiaA dynamics are coupled with changes in initial origin complexes leading to helicase loading. J. Biol. Chem. 284, 25038–25050. 10.1074/jbc.M109.002717 19632993 PMC2757208

[B55] Konieczny I. Doran K. S. Helinski D. R. Blasina A. (1997). Role of TrfA and DnaA proteins in origin opening during initiation of DNA replication of the broad host range plasmid RK2. J. Biol. Chem. 272, 20173–20178. 10.1074/jbc.272.32.20173 9242693

[B56] Konieczny I. Liberek K. (2002). Cooperative action of *Escherichia coli* ClpB protein and DnaK chaperone in the activation of a replication initiation protein. J. Biol. Chem. 277, 18483–18488. 10.1074/jbc.M107580200 11889118

[B57] Kowalczyk L. Rajewska M. Konieczny I. (2005). Positioning and the specific sequence of each 13-mer motif are critical for activity of the plasmid RK2 replication origin. Mol. Microbiol. 57, 1439–1449. 10.1111/j.1365-2958.2005.04770.x 16102011

[B58] Kowalski D. Eddy M. J. (1989). The DNA unwinding element: a novel, *cis*-acting component that facilitates opening of the *Escherichia coli* replication origin. EMBO J. 8, 4335–4344. 2556269 10.1002/j.1460-2075.1989.tb08620.x PMC401646

[B59] Krüger R. Filutowicz M. (2003). pi protein- and ATP-dependent transitions from ‘closed’ to ‘open’ complexes at the gamma *ori* of plasmid R6K. Nucleic Acids Res. 31, 5993–6003. 10.1093/nar/gkg809 14530447 PMC219486

[B60] Krüger R. Konieczny I. Filutowicz M. (2001). Monomer/dimer ratios of replication protein modulate the DNA strand-opening in a replication origin. J. Mol. Biol. 306, 945–955. 10.1006/jmbi.2000.4426 11237610

[B61] Kurokawa K. Nishida S. Emoto A. Sekimizu K. Katayama T. (1999). Replication cycle-coordinated change of the adenine nucleotide-bound forms of DnaA protein in *Escherichia coli*. EMBO J. 18, 6642–6652. 10.1093/emboj/18.23.6642 10581238 PMC1171727

[B62] Learn B. A. Um S. J. Huang L. McMacken R. (1997). Cryptic single-stranded-DNA binding activities of the phage lambda P and *Escherichia coli* DnaC replication initiation proteins facilitate the transfer of *E. coli* DnaB helicase onto DNA. Proc. Natl. Acad. Sci. U.S.A. 94, 1154–1159. 10.1073/pnas.94.4.1154 9037022 PMC19760

[B63] Learn B. Karzai A. W. McMacken R. (1993). Transcription stimulates the establishment of bidirectional lambda DNA replication *in vitro*. Cold Spring Harb. Symp. Quant. Biol. 58, 389–402. 10.1101/SQB.1993.058.01.046 7956053

[B64] Leonard A. C. Grimwade J. E. (2010). Initiation of DNA replication. EcoSal Plus 4. 10.1128/ecosalplus.4.4.1 26443786 PMC4236916

[B65] Leonard A. C. Grimwade J. E. (2015). The orisome: structure and function. Front. Microbiol. 6:545. 10.3389/fmicb.2015.00545 26082765 PMC4451416

[B66] Liu L. F. Wang J. C. (1987). Supercoiling of the DNA template during transcription. Proc. Natl. Acad. Sci. U.S.A. 84, 7024–7027. 10.1073/pnas.84.20.7024 2823250 PMC299221

[B67] Lu M. Campbell J. L. Boye E. Kleckner N. (1994). SeqA: a negative modulator of replication initiation in *E. coli*. Cell 77, 413–426. 10.1016/0092-8674(94)90156-2 8011018

[B68] Lu Y. B. Datta H. J. Bastia D. (1998). Mechanistic studies of initiator-initiator interaction and replication initiation. EMBO J. 17, 5192–5200. 10.1093/emboj/17.17.5192 9724655 PMC1170847

[B69] Mallory J. B. Alfano C. McMacken R. (1990). Host virus interactions in the initiation of bacteriophage lambda DNA replication. Recruitment of *Escherichia coli* DnaB helicase by lambda P replication protein. J. Biol. Chem. 265, 13297–13307. 2165499

[B70] Masukata H. Dasgupta S. Tomizawa J. (1987). Transcriptional activation of ColE1 DNA synthesis by displacement of the nontranscribed strand. Cell 51, 1123–1130. 10.1016/0092-8674(87)90598-8 2446775

[B71] Matsubara K. (1981). Replication control system in lambda dv. Plasmid 5, 32–52. 10.1016/0147-619X(81)90076-7 6452646

[B72] McGarry K. C. Ryan V. T. Grimwade J. E. Leonard A. C. (2004). Two discriminatory binding sites in the *Escherichia coli* replication origin are required for DNA strand opening by initiator DnaA-ATP. Proc. Natl. Acad. Sci. U.S.A. 101, 2811–2816. 10.1073/pnas.0400340101 14978287 PMC365702

[B73] Mensa-Wilmot K. Carroll K. McMacken R. (1989a). Transcriptional activation of bacteriophage lambda DNA replication *in vitro*: regulatory role of histone-like protein HU of *Escherichia coli*. EMBO J. 8, 2393–2402. 2529119 10.1002/j.1460-2075.1989.tb08369.x PMC401181

[B74] Mensa-Wilmot K. Seaby R. Alfano C. Wold M. C. Gomes B. McMacken R. (1989b). Reconstitution of a nine-protein system that initiates bacteriophage lambda DNA replication. J. Biol. Chem. 264, 2853–2861. 2536726

[B75] Merk A. Bartesaghi A. Banerjee S. Falconieri V. Rao P. Davis M. I.. (2016). Breaking Cryo-EM resolution barriers to facilitate drug discovery. Cell 165, 1698–1707. 10.1016/j.cell.2016.05.040 27238019 PMC4931924

[B76] Miller C. A. Beaucage S. L. Cohen S. N. (1990). Role of DNA superhelicity in partitioning of the pSC101 plasmid. Cell 62, 127–133. 10.1016/0092-8674(90)90246-B 2163765

[B77] Miller D. T. Grimwade J. E. Betteridge T. Rozgaja T. Torgue J. J. Leonard A. C. (2009). Bacterial origin recognition complexes direct assembly of higher-order DnaA oligomeric structures. Proc. Natl. Acad. Sci. U.S.A. 106, 18479–18484. 10.1073/pnas.0909472106 19833870 PMC2773971

[B78] Moore D. D. Denniston-Thompson K. Kruger K. E. Furth M. E. Williams B. G. Daniels D. L.. (1979). Dissection and comparative anatomy of the origins of replication of lambdoid phages. Cold Spring Harb. Symp. Quant. Biol. 43(Pt 1), 155–163. 10.1101/SQB.1979.043.01.022 157834

[B79] Mukherjee S. Patel I. Bastia D. (1985). Conformational changes in a replication origin induced by an initiator protein. Cell 43, 189–197. 10.1016/0092-8674(85)90023-6 3000600

[B80] Mukhopadhyay G. Carr K. M. Kaguni J. M. Chattoraj D. K. (1993). Open-complex formation by the host initiator, DnaA, at the origin of P1 plasmid replication. EMBO J. 12, 4547–4554. 8223464 10.1002/j.1460-2075.1993.tb06143.x PMC413885

[B81] Mukhopadhyay G. Chattoraj D. K. (1993). Conformation of the origin of P1 plasmid replication. Initiator protein induced wrapping and intrinsic unstacking. J. Mol. Biol. 231, 19–28. 10.1006/jmbi.1993.1253 8496963

[B82] Nakamura A. Wada C. Miki K. (2007). Structural basis for regulation of bifunctional roles in replication initiator protein. Proc. Natl. Acad. Sci. U.S.A. 104, 18484–18489. 10.1073/pnas.0705623104 18000058 PMC2141803

[B83] Neuwald A. F. Aravind L. Spouge J. L. Koonin E. V. (1999). AAA+: a class of chaperone-like ATPases associated with the assembly, operation, and disassembly of protein complexes. Genome Res. 9, 27–43. 9927482

[B84] Nguyen V. Q. Co C. Li J. J. (2001). Cyclin-dependent kinases prevent DNA re-replication through multiple mechanisms. Nature 411, 1068–1073. 10.1038/35082600 11429609

[B85] Nievera C. Torgue J. J. Grimwade J. E. Leonard A. C. (2006). SeqA blocking of DnaA-*oriC* interactions ensures staged assembly of the *E. coli* pre-RC. Mol. Cell 24, 581–592. 10.1016/j.molcel.2006.09.016 17114060 PMC1939805

[B86] Odegrip R. Schoen S. Haggard-Ljungquist E. Park K. Chattoraj D. K. (2000). The interaction of bacteriophage P2 B protein with *Escherichia coli* DnaB helicase. J. Virol. 74, 4057–4063. 10.1128/JVI.74.9.4057-4063.2000 10756017 PMC111919

[B87] O'Donnell M. (2006). Replisome architecture and dynamics in *Escherichia coli*. J. Biol. Chem. 281, 10653–10656. 10.1074/jbc.R500028200 16421093

[B88] Ogawa T. Tomizawa J. (1968). Replication of bacteriophage DNA. I. Replication of DNA of lambda phage defective in early functions. J. Mol. Biol. 38, 217–225. 10.1016/0022-2836(68)90407-5 4941475

[B89] Osipiuk J. Georgopoulos C. Zylicz M. (1993). Initiation of lambda DNA replication. The *Escherichia coli* small heat shock proteins, DnaJ and GrpE, increase DnaK's affinity for the lambda P protein. J. Biol. Chem. 268, 4821–4827. 8444859

[B90] Ozaki S. Katayama T. (2009). DnaA structure, function, and dynamics in the initiation at the chromosomal origin. Plasmid 62, 71–82. 10.1016/j.plasmid.2009.06.003 19527752

[B91] Ozaki S. Katayama T. (2012). Highly organized DnaA-oriC complexes recruit the single-stranded DNA for replication initiation. Nucleic Acids Res. 40, 1648–1665. 10.1093/nar/gkr832 22053082 PMC3287180

[B92] Ozaki S. Kawakami H. Nakamura K. Fujikawa N. Kagawa W. Park S. Y.. (2008). A common mechanism for the ATP-DnaA-dependent formation of open complexes at the replication origin. J. Biol. Chem. 283, 8351–8362. 10.1074/jbc.M708684200 18216012

[B93] Park K. Chattoraj D. K. (2001). DnaA boxes in the P1 plasmid origin: the effect of their position on the directionality of replication and plasmid copy number. J. Mol. Biol. 310, 69–81. 10.1006/jmbi.2001.4741 11419937

[B94] Park K. Mukhopadhyay S. Chattoraj D. K. (1998). Requirements for and regulation of origin opening of plasmid P1. J. Biol. Chem. 273, 24906–24911. 10.1074/jbc.273.38.24906 9733797

[B95] Paulsson J. Chattoraj D. K. (2006). Origin inactivation in bacterial DNA replication control. Mol. Microbiol. 61, 9–15. 10.1111/j.1365-2958.2006.05229.x 16824091

[B96] Petojevic T. Pesavento J. J. Costa A. Liang J. Wang Z. Berger J. M.. (2015). Cdc45 (cell division cycle protein 45) guards the gate of the Eukaryote Replisome helicase stabilizing leading strand engagement. Proc. Natl. Acad. Sci. U.S.A. 112, E249–E258. 10.1073/pnas.1422003112 25561522 PMC4311868

[B97] Polaczek P. Kwan K. Campbell J. L. (1998). Unwinding of the *Escherichia coli* origin of replication (*oriC*) can occur in the absence of initiation proteins but is stabilized by DnaA and histone-like proteins IHF or HU. Plasmid 39, 77–83. 10.1006/plas.1997.1328 9473448

[B98] Rahmouni A. R. Wells R. D. (1989). Stabilization of Z DNA *in vivo* by localized supercoiling. Science 246, 358–363. 10.1126/science.2678475 2678475

[B99] Rajewska M. Kowalczyk L. Konopa G. Konieczny I. (2008). Specific mutations within the AT-rich region of a plasmid replication origin affect either origin opening or helicase loading. Proc. Natl. Acad. Sci. U.S.A. 105, 11134–11139. 10.1073/pnas.0805662105 18685104 PMC2516259

[B100] Richardson T. T. Harran O. Murray H. (2016). The bacterial DnaA-trio replication origin element specifies single-stranded DNA initiator binding. Nature 534, 412–416. 10.1038/nature17962 27281207 PMC4913881

[B101] Rozgaja T. A. Grimwade J. E. Iqbal M. Czerwonka C. Vora M. Leonard A. C. (2011). Two oppositely oriented arrays of low-affinity recognition sites in oriC guide progressive binding of DnaA during *Escherichia coli* pre-RC assembly. Mol. Microbiol. 82, 475–488. 10.1111/j.1365-2958.2011.07827.x 21895796 PMC3192301

[B102] Ryan V. T. Grimwade J. E. Camara J. E. Crooke E. Leonard A. C. (2004). *Escherichia coli* prereplication complex assembly is regulated by dynamic interplay among Fis, IHF and DnaA. Mol. Microbiol. 51, 1347–1359. 10.1046/j.1365-2958.2003.03906.x 14982629

[B103] Saito H. Uchida H. (1977). Initiation of the DNA replication of bacteriophage lambda in *Escherichia coli* K12. J. Mol. Biol. 113, 1–25. 10.1016/0022-2836(77)90038-9 328896

[B104] Saxena R. Vasudevan S. Patil D. Ashoura N. Grimwade J. E. Crooke E. (2015). Nucleotide-induced conformational changes in *Escherichia coli* DnaA protein are required for bacterial ORC to Pre-RC conversion at the chromosomal origin. Int. J. Mol. Sci. 16, 27897–27911. 10.3390/ijms161126064 26610483 PMC4661922

[B105] Schaper S. Messer W. (1995). Interaction of the initiator protein DnaA of *Escherichia coli* with its DNA target. J. Biol. Chem. 270, 17622–17626. 10.1074/jbc.270.29.17622 7615570

[B106] Schnos M. Inman R. B. (1970). Position of branch points in replicating lambda DNA. J. Mol. Biol. 51, 61–73. 10.1016/0022-2836(70)90270-6 5481280

[B107] Schnos M. Zahn K. Inman R. B. Blattner F. R. (1988). Initiation protein induced helix destabilization at the lambda origin: a prepriming step in DNA replication. Cell 52, 385–395. 10.1016/S0092-8674(88)80031-X 2830983

[B108] Scholefield G. Errington J. Murray H. (2012). Soj/ParA stalls DNA replication by inhibiting helix formation of the initiator protein DnaA. EMBO J. 31, 1542–1555. 10.1038/emboj.2012.6 22286949 PMC3321191

[B109] Sharma R. Kachroo A. Bastia D. (2001). Mechanistic aspects of DnaA-RepA interaction as revealed by yeast forward and reverse two-hybrid analysis. EMBO J. 20, 4577–4587. 10.1093/emboj/20.16.4577 11500384 PMC125567

[B110] Shibata T. Nishinaka T. Mikawa T. Aihara H. Kurumizaka H. Yokoyama S.. (2001). Homologous genetic recombination as an intrinsic dynamic property of a DNA structure induced by RecA/Rad51-family proteins: a possible advantage of DNA over RNA as genomic material. Proc. Natl. Acad. Sci. U.S.A. 98, 8425–8432. 10.1073/pnas.111005198 11459985 PMC37453

[B111] Simmons L. A. Felczak M. Kaguni J. M. (2003). DnaA Protein of *Escherichia coli*: oligomerization at the *E. coli* chromosomal origin is required for initiation and involves specific N-terminal amino acids. Mol. Microbiol. 49, 849–858. 10.1046/j.1365-2958.2003.03603.x 12864864

[B112] Skarstad K. Baker T. A. Kornberg A. (1990). Strand separation required for initiation of replication at the chromosomal origin of *E. coli* is facilitated by a distant RNA–DNA hybrid. EMBO J. 9, 2341–2348. 1694129 10.1002/j.1460-2075.1990.tb07406.x PMC551962

[B113] Skarstad K. Katayama T. (2013). Regulating DNA replication in bacteria. Cold Spring Harb. Perspect. Biol. 5:a012922. 10.1101/cshperspect.a012922 23471435 PMC3683904

[B114] Speck C. Messer W. (2001). Mechanism of origin unwinding: sequential binding of DnaA to double- and single-stranded DNA. EMBO J. 20, 1469–1476. 10.1093/emboj/20.6.1469 11250912 PMC145534

[B115] Stenzel T. T. Patel P. Bastia D. (1987). The integration host factor of *Escherichia coli* binds to bent DNA at the origin of replication of the plasmid pSC101. Cell 49, 709–717. 10.1016/0092-8674(87)90547-2 3555843

[B116] Stepankiw N. Kaidow A. Boye E. Bates D. (2009). The right half of the *Escherichia coli* replication origin is not essential for viability, but facilitates multi-forked replication. Mol. Microbiol. 74, 467–479. 10.1111/j.1365-2958.2009.06877.x 19737351 PMC2967437

[B117] Sutton M. D. Carr K. M. Vicente M. Kaguni J. M. (1998). *Escherichia coli* DnaA protein. The N-terminal domain and loading of DnaB helicase at the *E. coli* chromosomal origin. J. Biol. Chem. 273, 34255–34262. 10.1074/jbc.273.51.34255 9852089

[B118] Szambowska A. Pierechod M. Wegrzyn G. Glinkowska M. (2011). Coupling of transcription and replication machineries in lambda DNA replication initiation: evidence for direct interaction of *Escherichia coli* RNA polymerase and the lambdaO protein. Nucleic Acids Res. 39, 168–177. 10.1093/nar/gkq752 20833633 PMC3017604

[B119] Teter B. Goodman S. D. Galas D. J. (2000). DNA bending and twisting properties of integration host factor determined by DNA cyclization. Plasmid 43, 73–84. 10.1006/plas.1999.1443 10610821

[B120] Thomas R. Bertani L. E. (1964). On the control of the replication of temperate bacteriophages superinfecting immune hosts. Virology 24, 241–253. 14227027 10.1016/0042-6822(64)90163-1

[B121] Tomizawa J. Itoh T. Selzer G. Som T. (1981). Inhibition of ColE1 RNA primer formation by a plasmid-specified small RNA. Proc. Natl. Acad. Sci. U.S.A. 78, 1421–1425. 10.1073/pnas.78.3.1421 6165011 PMC319142

[B122] Toukdarian A. Helinski D. R. Perri S. (1996). The plasmid RK2 initiation protein binds to the origin of replication as a monomer. J. Biol. Chem. 271, 7072–7078. 10.1074/jbc.271.12.7072 8636140

[B123] Tsurimoto T. Matsubara K. (1981). Purified bacteriophage lambda O protein binds to four repeating sequences at the lambda replication origin. Nucleic Acids Res. 9, 1789–1799. 10.1093/nar/9.8.1789 6264392 PMC326803

[B124] Vlijm R. v. d. Torre J. Dekker C. (2015). Counterintuitive DNA sequence dependence in supercoiling-induced DNA melting. PLoS ONE 10:e0141576. 10.1371/journal.pone.0141576 26513573 PMC4625975

[B125] Wegrzyn K. Fuentes-Perez M. E. Bury K. Rajewska M. Moreno-Herrero F. Konieczny I. (2014). Sequence-specific interactions of Rep proteins with ssDNA in the AT-rich region of the plasmid replication origin. Nucleic Acids Res. 42, 7807–7818. 10.1093/nar/gku453 24838560 PMC4081077

[B126] Weigel C. Messer W. Preiss S. Welzeck M. Morigen Boye, E. (2001). The sequence requirements for a functional *Escherichia coli* replication origin are different for the chromosome and a minichromosome. Mol. Microbiol. 40, 498–507. 10.1046/j.1365-2958.2001.02409.x 11309131

[B127] Wells R. D. Larson J. E. Grant R. C. Shortle B. E. Cantor C. R. (1970). Physicochemical studies on polydeoxyribonucleotides containing defined repeating nucleotide sequences. J. Mol. Biol. 54, 465–497. 10.1016/0022-2836(70)90121-X 5492018

[B128] Westmoreland B. C. Szybalski W. Ris H. (1969). Mapping of deletions and substitutions in heteroduplex DNA molecules of bacteriophage lambda by electron microscopy. Science 163, 1343–1348. 10.1126/science.163.3873.1343 5765116

[B129] Wickner S. Gottesman S. Skowyra D. Hoskins J. McKenney K. Maurizi M. R. (1994). A molecular chaperone, ClpA, functions like DnaK and DnaJ. Proc. Natl. Acad. Sci. U.S.A. 91, 12218–12222. 10.1073/pnas.91.25.12218 7991609 PMC45408

[B130] Wickner S. Hoskins J. McKenney K. (1991). Monomerization of RepA dimers by heat shock proteins activates binding to DNA replication origin. Proc. Natl. Acad. Sci. U.S.A. 88, 7903–7907. 10.1073/pnas.88.18.7903 1896443 PMC52413

[B131] Woelker B. Messer W. (1993). The structure of the initiation complex at the replication origin, *oriC*, of *Escherichia coli*. Nucleic Acids Res. 21, 5025–5033. 10.1093/nar/21.22.5025 8255756 PMC310613

[B132] Womble D. D. Rownd R. H. (1986). Regulation of lambda dv plasmid DNA replication. A quantitative model for control of plasmid lambda dv replication in the bacterial cell division cycle. J. Mol. Biol. 191, 367–382. 10.1016/0022-2836(86)90133-6 2950236

[B133] Yardimci H. Walter J. C. (2014). Prereplication-complex formation: a molecular double take? Nat. Struct. Mol. Biol. 21, 20–25. 10.1038/nsmb.2738 24389553

[B134] Zahn K. Blattner F. R. (1987). Direct evidence for DNA bending at the lambda replication origin. Science 236, 416–422. 10.1126/science.2951850 2951850

[B135] Zorman S. Seitz H. Sclavi B. Strick T. R. (2012). Topological characterization of the DnaA-*oriC* complex using single-molecule nanomanipuation. Nucleic Acids Res. 40, 7375–7383. 10.1093/nar/gks371 22581769 PMC3424547

[B136] Zylicz M. Ang D. Liberek K. Georgopoulos C. (1989). Initiation of lambda DNA replication with purified host- and bacteriophage-encoded proteins: the role of the DnaK, DnaJ and GrpE heat shock proteins. EMBO J. 8, 1601–1608. 2527744 10.1002/j.1460-2075.1989.tb03544.x PMC400992

[B137] Zzaman S. Abhyankar M. M. Bastia D. (2004a). Reconstitution of F factor DNA replication *in vitro* with purified proteins. J. Biol. Chem. 279, 17404–17410. 10.1074/jbc.M400021200 14973139

[B138] Zzaman S. Bastia D. (2005). Oligomeric initiator protein-mediated DNA looping negatively regulates plasmid replication *in vitro* by preventing origin melting. Mol. Cell 20, 833–843. 10.1016/j.molcel.2005.10.037 16364910

[B139] Zzaman S. Reddy J. M. Bastia D. (2004b). The DnaK-DnaJ-GrpE chaperone system activates inert wild type pi initiator protein of R6K into a form active in replication initiation. J. Biol. Chem. 279, 50886–50894. 10.1074/jbc.M407531200 15485812

